# Impairment of novelty-related theta oscillations and P3a in never medicated first-episode psychosis patients

**DOI:** 10.1038/s41537-021-00146-3

**Published:** 2021-02-26

**Authors:** Rodolfo Solís-Vivanco, Alejandra Mondragón-Maya, Francisco Reyes-Madrigal, Camilo de la Fuente-Sandoval

**Affiliations:** 1 Laboratory of Neuropsychology, Instituto Nacional de Neurología y Neurocirugía, Mexico City, Mexico; 2 Faculty of Psychology, Universidad Nacional Autónoma de México, Mexico City, Mexico; 3 Faculty of Higher Studies Iztacala, Universidad Nacional Autónoma de México, Mexico City, Mexico; 4 Laboratory of Experimental Psychiatry, Instituto Nacional de Neurología y Neurocirugía, Mexico City, Mexico; 5 Neuropsychiatry Department, Instituto Nacional de Neurología y Neurocirugía, Mexico City, Mexico

**Keywords:** Biomarkers, Psychosis

## Abstract

We explored the neurophysiological activity underlying auditory novelty detection in antipsychotic-naive patients with a first episode of psychosis (FEP). Fifteen patients with a non-affective FEP and 13 healthy controls underwent an active involuntary attention task along with an EEG acquisition. Time-frequency representations of power, phase locking, and fronto-parietal connectivity were calculated. The P3a event-related potential was extracted as well. Compared to controls, the FEP group showed reduced theta phase-locking and fronto-parietal connectivity evoked by deviant stimuli. Also, the P3a amplitude was significantly reduced. Moreover, reduced theta connectivity was associated with more severe negative symptoms within the FEP group. Reduced activity (phase-locking and connectivity) of novelty-related theta oscillations, along with P3a reduction, may represent a failure to synchronize large-scale neural populations closely related to fronto-parietal attentional networks, and might be explored as a potential biomarker of disease severity in patients with emerging psychosis, given its association with negative symptoms.

## Introduction

Schizophrenia is a chronic and disabling psychotic disorder associated with cognitive impairment^1^. The importance of cognitive functioning in schizophrenia has led to explore the nature of such deficits using different approaches. The neurophysiological approach aims to comprehend the information processing that underlie cognitive functions, by analyzing the brain electrical activity during experimental situations^2^. Event related potentials (ERP), one of the most frequent methods from this approach, consist of voltage changes associated with stimuli or events, reflecting the way the brain processes the information related to them^3,4^.

Detection of novel, yet unattended events is highly relevant for everyday life, and ERP studies exploring the P3a wave have shown that this process is frequently impaired in different neuropsychiatric diseases^5^. The P3a is an ERP involved in automatic orienting of attention appearing at frontocentral scalp regions around 250–300 ms after the presentation of deviant or unexpected stimuli^6^. The classical paradigms used to obtain the P3a may involve three types of stimuli: standard, deviant (target) and novel (non–target) stimuli^7^, or standard and deviant stimuli, with the latter showing a difference in any physical feature (e.g. pitch or duration) that is irrelevant for the task. The task can be recorded under passive or resting conditions, or under active performance of the subject, who must usually discriminate between standard and target stimuli during a stimulation block including unexpected novel stimuli. The latter stimuli will evoke the P3a. Reduced amplitude of this ERP has been consistently reported in ultra-high risk for psychosis (UHR), first-episode psychosis (FEP), and chronic schizophrenia patients, with most of the research being performed with medicated patients under passive conditions^8–15^. Two reports described reduced P3a using active paradigms in medicated patients^16,17^, and three studies showed P3a deficits in unmedicated patients under passive tasks^18–20^. Nevertheless, to our knowledge, no reports of P3a obtained with active paradigms in unmedicated patients have been performed (for a review on these studies see Justo-Guillén et al.^5^).

Recently, alternative data analysis obtained from ERP paradigms have been widely used, such as brain oscillations derived from time-frequency analyses. This approach complements the atypical processing underlying the ERP differences observed in clinical populations^21^. Altogether, these neurophysiological measures have been proposed as biomarkers of cognitive dysfunction in neuropsychiatric disorders^22^. Among these, time-frequency analysis provides two measures additional to the ERP approach: the power of a single trial induced by the stimulus, reflecting the EEG response magnitude to each stimulus, and the intertrial coherence or phase-locking factor (PLF), which reflects the rhythmic response consistency across repetitive stimulation^23^. Under this approach, some auditory and visual ERP—like P3a—show a maximal underlying activity in the theta frequency band (4–7 Hz)^24^.

There is evidence regarding abnormal theta oscillations in patients with schizophrenia, including increased amplitude at rest or under passive conditions^25–28^ and reduced activity for targets during active paradigms^21,29–31^. According to those studies, theta activity is impaired in schizophrenia possibly due to connectivity networks disruption at different scales, which in turn are reflected as defective performance. This approach suggests that a core pathophysiological feature of schizophrenia consists of a functional disconnection syndrome, in which theta oscillations are strongly implicated^32^.

To our knowledge, novelty detection has been studied in patients with psychosis mainly under the ERP approach. Moreover, the majority of those reports have studied chronic patients undergoing antipsychotic medication or under passive conditions. Thus, our aim in this study was to explore the theta oscillations underlying active auditory novelty detection in unmedicated patients with a FEP. In addition, since auditory novel stimuli have been shown to modulate fronto-parietal attention networks as revealed by theta oscillations^33^, we also explored the time-frequency connectivity between frontal and parietal regions after novelty detection. Finally, we explored whether abnormal theta activity is associated with symptom severity in these patients.

## Results

### Demographic and clinical data

Table 1 shows the demographic and clinical data in both groups. The mean age across all participants was 23.9 ± 6 years, with no significant differences between groups. Women represented 24% of the total sample, again with no significant differences between groups. Most of the patients were diagnosed with schizophrenia (73%) and schizophreniform disorder (20%). As expected, FEP patients showed less school years than healthy controls (HC). Also, the parents’ education in the FEP was significantly lower, thus we adjusted all our comparisons between groups by the mean education of both parents.

**Table 1:** Demographic, clinical, and behavioral variables.

	FEP Mean (SD)	HC Mean (SD)	Statistic	*p*
Age (years)	25.93 (7.84)	22.46 (2.33)	t=1.54	0.14
Gender (female/male)	5/10	2/11	*X*^2^=0.27	0.39
Education (years)	11.21 (3.31)	15.92 (1.19)	t=4.84	<0.001
Parents’ mean education (years)	8.75 (3.61)	13.96 (3.06)	t=3.93	0.001
Diagnosis, *N* (%)				
Schizophrenia	11 (73)	–	–	–
Schizofreniform disorder	3 (20)	–	–	–
Brief psychotic disorder	1 (7)	–	–	–
PANSS				
Positive	26.14 (4.50)	–	–	–
Negative	24.93 (4.39)	–	–	–
General Psychopathology	50.71 (4.43)	–	–	–
Total score	101.93 (9.99)	–	–	–
MCCB- total score (t score)	28.20 (10.27)	48.77 (7.65)	t=5.93	<0.001
Task performance				
RT (ms)				
Deviant	752.81 (89.68)	736.90 (60.01)	F=0.04^1^	0.85
Frequent	694.36 (87.48)	692.57 (54.48)		
Hit rate				
Deviant	0.52 (0.20)	0.76 (0.12)	F=8.77^1^	0.007
Frequent	0.59 (0.19)	0.85 (0.09)		

*HT* Hit rate, *MCCB* MATRICS Cognitive Consensus Battery, *PANSS* Positive and Negative Symptoms scale, *RT* Reaction time.

^1^Repeated measures ANOVA, adjusted by parents’ education.

### Experimental paradigm- Behavior

**Fig. 1:**
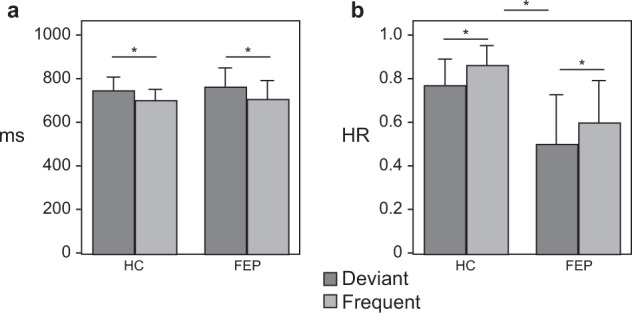
Behavioral performance in the experimental paradigm. **a** Reaction times revealed delayed responses for deviant vs. frequent tones, with no differences between groups. **b** Hit rates showed reduced performance for deviant compared with frequent tones in both groups. The FEP group also showed reduced general performance along the task. The error bars represent the standard deviation. *p < 0.01.

RT were significantly longer for deviant compared with frequent tones (F_(1,25)_ = 32.2, *p* < 0.001). No significant differences were found between groups for either tone type (F_(1,25)_ = 0.04, *p* = 0.85; Fig. 1a).

Deviant tones showed lower HR compared with frequent tones (F_(1,25)_ = 10.41, *p* = 0.003). This time, the FEP group showed significantly lower HR compared with the HC group (F_(1,25)_ = 8.76, *p* = 0.007), regardless of tone type (F_(1,25)_ = 0.14, *p* = 0.72; Fig. 1b).

### Time and phase-locked theta oscillations

**Fig. 2:**
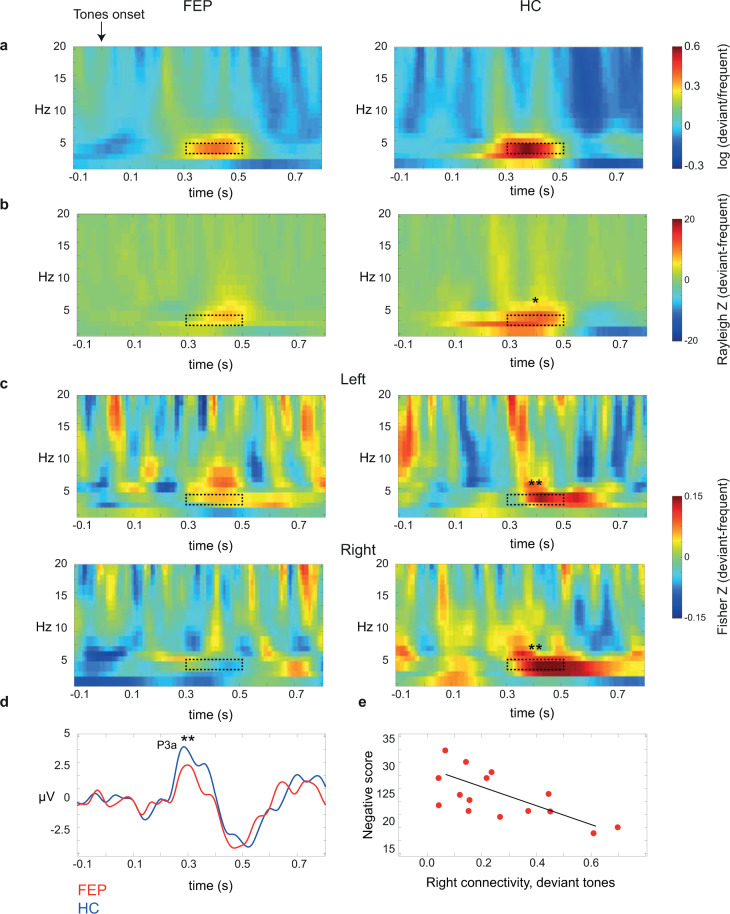
Theta oscillations differences (deviant - frequent) in each group. **a** TFR of power. **b** PLF. **c** Left (F7-P7) and right (F8-P8) fronto-parietal connectivity. **d** ERP (P3a). **e** Lower right theta connectivity for deviant tones was associated with higher scores of negative symptoms (*p* = 0.007). Dashed rectangles indicate time-frequency ranges of interest. Asterisks indicate significant differences between tone types (only present in the HC group). *p < 0.05, **p ≤ 0.001.

Table 2 shows descriptive data for power, PLF, connectivity, and P3a values in each group. Figure 2a shows the TFR of power for each group. As expected, power was increased for deviant compared with frequent tones (F_(1,24)_ = 6.32, *p* = 0.018). Nevertheless, there were no significant differences between groups (F_(1,24)_ = 0.23, *p* = 0.63), nor a significant interaction of group by tone (F_(1,24)_ = 2.47, *p* = 0.13).

**Table 2:** Neurophysiological variables in each group.

	FEP Mean (SD)		HC Mean (SD)		F^1^	*p*
	Deviant	Frequent	Deviant	Frequent		
Theta						
Power (log(power))	16.53 (8.92)	12.09 (7.09)	14.66 (6.90)	8.92 (3.89)	2.47	0.13
PLF (Rayleigh Z)	7.80 (6.07)	2.76 (2.18)	12.66 (8.04)	3.22 (3.25)	4.76	0.04
Connectivity (Fisher Z)						
Left	0.27 (0.20)	0.22 (0.13)	0.32 (0.16)	0.23 (0.11)	7.31	0.01
Right	0.22 (0.20)	0.22 (0.11)	0.35 (0.14)	0.22 (0.12)		
P3a (µV)	2.96 (1.26)	1.48 (0.86)	4.28 (2.16)	1.43 (1.54)	9.47	0.006

*PLF* Phase-locking factor.

^1^Interaction of group by tones from repeated measures ANOVA, adjusted by parents’ education and number of trials.

PLF of theta oscillations revealed enhanced phase adjustment for deviant compared with frequent tones (F_(1,24)_ = 4.20, *p* = 0.05) and a reduced general phase alignment in the FEP group compared with the HC group (F_(1,24)_ = 5.16, *p* = 0.03). Also, a significant interaction between group and tone was found (F_(1,24)_ = 4.76 *p* = 0.04). Post hoc comparisons revealed significant reduced phase locking for the deviant tones in the FEP group compared with HC (MD = −8.98, *p* = 0.02), but no differences between groups for the frequent tones (mean difference (MD) = 0.10, *p* = 0.95). Also, while the HC group showed increased PLF values for deviant vs. frequent tones (MD = 11.72, *p* < 0.001), no significant differences were found in the FEP group between tones (MD = 2.85, *p* = 0.24; Fig. 2b; Table 2). In addition, while PLF of deviant tones were strongly associated with the corresponding RT in the HC group (*r* = 0.77, *p* = 0.002), that was not the case for the FEP group (*r* = −0.48, *p* = 0.07).

### Theta connectivity

We found increased fronto-parietal connectivity for deviant compared with frequent tones (F_(1,24)_ = 13.87, *p* = 0.001). Also, a significant interaction of group by tone was found (F_(1,24)_ = 7.31, *p* = 0.01). Post hoc comparison revealed significant increases of connectivity for deviant compared to frequent tones in the HC group (MD = 0.13, *p* = 0.001), but this effect was not present in the FEP group (MD = 0.01, *p* = 0.72; Fig. 2c; Table 2). Across participants, theta connectivity was significantly associated with PLF (*r* = 0.49, *p* = 0.008) and P3a (*r* = 0.43, *p* = 0.02), but not with theta power (*r* = 0.15, *p* = 0.44).

### P3a

A significant effect of tone was observed (F_(1,24)_ = 6.34, *p* = 0.02), with higher voltage for deviant compared to frequent tones. Again, a significant interaction of group by tone was found (F_(1,24)_ = 9.47, *p* = 0.006). Post hoc comparisons revealed a significant P3a increase for deviant tones in the HC group (MD = 3.33, *p* < 0.001), but not in the FEP group (MD = 0.82, *p* = 0.10; Fig. 2d; Table 2). Across participants, the P3a amplitude was significantly associated with theta PLF (*r* = 0.63, *p* < 0.001). P3a latencies were not significantly different between groups (t_(27)_ = 0.75, *p* = 0.46).

### Theta oscillations, P3a, and clinical features

Associations between theta PLF, theta connectivity, and P3a, all for deviant tones, and the positive and negative syndrome scale (PANSS) scores were explored within the FEP group. An inverse significant correlation was found between PLF and negative symptoms (*r* = −0.53, *p* = 0.05). Nevertheless, this correlation did not survive Bonferroni correction (alpha = 0.0125). The P3a did not show significant associations with any score. In the case of theta connectivity, a significant inverse correlation was found between right fronto-parietal values (F8-P8) and negative symptoms (*r* = −0.68, *p* = 0.007; Fig. 2e). A subsequent exploration of the specific negative symptoms that were inversely related to theta connectivity revealed (non-corrected) significance for blunted affect (N1, *r* = −0.54, *p* = 0.04), emotional withdrawal (N2, *r* = −0.59, *p* = 0.02), poor rapport (N3, *r* = −0.61, *p* = 0.01), and lack of spontaneity (N6, *r* = −0.55, *p* = 0.04).

No significant correlations were found between theta PLF, theta connectivity, and P3a, with the MATRICS Cognitive Consensus Battery (MCCB) total scores. In order to discard that the MCCB total score might hide potential associations with specific cognitive domains, we explored correlations with the Attention/Vigilance, Speed of Processing, and Working Memory domains. Nevertheless, no significant associations were found (all *r* ≤ 0.26, all *p* ≥ 0.34).

## Discussion

The aim of this study was to explore the underlying neurophysiological dynamics of novelty detection in antipsychotic naïve FEP patients. Our main results were significant reductions of both P3a and phase resetting of theta oscillations evoked by deviant auditory stimuli in the group of patients. In addition, reduced fronto-parietal theta connectivity elicited by deviance was found. In addition, right theta connectivity was inversely associated with severity of negative symptoms.

To our knowledge, this is the first study exploring the neurophysiological correlates of auditory novelty detection under an active paradigm in never-medicated patients. Some coincidences and discrepancies are found with other studies. In the present study, we did not observe differences in theta total power across groups, whilst phase resetting of theta activity was found to be reduced for deviant stimuli in the FEP group, both between groups and compared to frequent stimuli within this group. Therefore, a diminished reaction to unexpected events can be concluded in these patients, but also a general reduction of phase resetting after sensory reception. While no previous studies reported abnormal phase locking of theta oscillations evoked by novel stimuli in FEP patients, Doege et al.^34^ reported decreased PLF of slow oscillations (particularly delta) in a P300 paradigm in patients with established schizophrenia, which may be compatible with our findings. On the other hand, our results differ from Bates et al.^29^, who found decreased power of slow oscillations after detection of target stimuli in chronic medicated patients, but are similar in terms of reduced phase resetting. The authors concluded not only an impaired evoked synchronization of slow waves after target detection, but also reduced activity of non-time locked (induced) slow oscillations in these patients. Since a similar reduction of power has been reported in other studies with chronic patients^35^, we hypothesize that patients with recent onset psychosis are mainly affected by the timing of neural synchronization of large-scale networks evoked by bottom-up processes, rather than a general failure to recruit them in response to novel stimuli. Importantly, such timing failure seems to be present for both expected and unexpected stimuli, indicating a general impairment on neural synchronization for attentional and perceptual processing. In accordance, as the illness progresses, failures in neural recruitment might become more evident, as revealed by reduced both evoked (phase resetting) and induced theta power. This hypothesis should be explored with a longitudinal study comparing recent psychosis onset versus established schizophrenia in the same sample of patients, since dissimilar results in theta power and phase resetting have been reported as well in chronic patients using oddball paradigms^36^.

It should be noted that in our experimental paradigm deviant stimuli contained physical properties both relevant (duration) and not relevant for the task (pitch change). Such type of deviance includes a first involuntary attentional capture enhanced by pitch and then an attentional reorientation to the relevant component of the task (duration). Therefore, attentional reorientation might be included, at least in part, in the observed evoked theta activity. For instance, attentional reorientation can be constrained to the theta cycle^37^ and phase resetting of theta oscillations have been shown to increase after attentional reorientation^38^. Therefore, the theta oscillations and P3a anomalies observed in FEP patients might also reflect a general dysfunction within the attention reorienting system, which includes dorsal and ventral attention networks (DAN and VAN, respectively). DAN corresponds to top-down mechanisms, whilst VAN is associated with bottom-up processing^33,39^.

The connectivity analysis showed reduced reactivity to deviant tones in the FEP group, which is compatible with a general dysfunction of the attention reorienting system in patients, as mentioned before. Interestingly, only right connectivity was sensible to negative symptoms. This finding, although unexpected, is particularly striking, since VAN activation is predominantly right-sided^39^. Specifically, significant associations between reduced connectivity and negative symptoms comprised blunted affect, emotional withdrawal, poor rapport, and lack of spontaneity. All of these symptoms—but emotional withdrawal—comprise the factor 1 “Expressive deficits” proposed by Liemburg et al.^40^. These authors observed that Expressive deficits correspond to the core negative symptoms of schizophrenia, in which loss of initiative is predominant. We suggest that such deficits could be closely associated with a reduced responsivity to environmental stimulation, particularly the novel one, which in turn may be displayed as lack of initiative or reduced behavioral/emotional expression in these patients. Our suggestion relies on the functional role of the VAN, closely related to bottom-up detection of unexpected, yet relevant stimuli. This is consistent with Javitt^41^, who proposed that the failure of bottom-up mechanisms observed in schizophrenia may impair patients’ ability to detect salient and potentially relevant stimuli, thus affecting their chance of voluntarily focusing and responding to significant environmental events.

We found a significant amplitude reduction of P3a in FEP patients. Our results are consistent with previous reports with unmedicated patients^18,19^, though here we demonstrate the presence of P3a deficits under an active paradigm, which had not been tested before. Moreover, our results match with an fMRI-ERP study that showed reduced P3a after unexpected stimuli in schizophrenia. That study also showed a close relationship between this ERP and the activation of the VAN^42^. In the same line, Laurens et al.^43^ performed an fMRI-ERP study to explore the attention orienting response to salient novel stimulus in schizophrenia. Relative to healthy controls, they found hypoactivity during novel stimuli processing, specifically at right cortical regions involved in the VAN. Given that our patients were antipsychotic naïve, while the ones in the studies of Laurens et al.^43^ and Wynn et al.^42^ were not, a positive or negative effect of medication over novelty detection in psychosis can be discarded. As our group has observed, many studies have shown that P3a is sensitive to psychosis, mainly in the auditory modality and using the classical paradigm for novelty detection^5^. The present study replicated such findings with unmedicated patients, thus supporting the proposal of P3a as a promising biomarker for psychosis.

Interestingly, the significant effects of PLF and P3a observed in FEP patients are consistently related. PLF values showed a disturbed general phase adjustment regardless of the type of stimuli. Moreover, a phase resetting reduction to deviant stimuli was particularly evident in patients. These findings might indicate impaired neuronal synchronization as a feature of psychosis, affecting the novelty detection response, which in turn is represented as a diminished P3a.

We did not find a significant association between P3a or theta oscillations and MCCB scores. This lack of relation may indicate different stages of information processing between EEG oscillations evoked by sensory stimulation and more top-down guided tasks as reflected by MCCB scores. This result highlights the importance of cognitive assessment beyond traditional neuropsychological testing, and calls for a complementary approach, especially when in search for neurocognitive biomarkers of psychosis.

Among the advantages of our study, this study explored novelty detection under an active paradigm in never-medicated patients. To our knowledge, this is the first study with this approach. Also, time-frequency windows of interest were based on data outcomes across all participants, rather than on a priori selection based on previous reports in schizophrenia.

Our study has limitations and opens questions for future research. The small sample size makes it difficult to establish strong generalizations, so upcoming studies might include the P3a and theta oscillations as part of their protocols, especially for between-sites collaborations. Also, in this first exploratory study, we included a reduced number of EEG channels, which limits the possibility of identifying the source of the affected theta activity in these patients. Specifically, the reduced fronto-parietal connectivity evoked by deviant stimuli should be further explored at source level with techniques with stronger spatial resolution, such as high-density EEG or magnetoencephalography. Also, phase resetting of high frequency oscillations (i.e. gamma) have shown to be impaired in recent onset psychosis^44^, so they should be explored in unmedicated FEP patients. Finally, our results with P3a and evoked theta activity should be explored in patients with chronic psychosis in order to know whether this impairment worsens along illness progression or remains stable, as shown with other cognitive domains in this clinical population^45^.

In conclusion, we found significant reductions of theta oscillations (phase resetting and connectivity) and P3a amplitudes after detection of novel stimuli in never-medicated patients with a FEP. Such reductions might represent impaired synchronization of large-scale neural populations closely related to fronto-parietal attention networks and might be explored as a potential biomarker of disease severity in these patients, given its association with negative symptoms.

## Methods

### Participants

Fifteen patients in their first non-affective psychosis episode, according to the Structured Clinical Interview for DSM-IV^46^ were recruited from the inpatient psychiatric service, the emergency department, and the Adolescent Program of Neuropsychiatric and Imaging Study (PIENSA) of the Instituto Nacional de Neurología y Neurocirugía (INNN) of Mexico. FEP participants were interviewed and diagnosed by at least one psychiatrist specialized in psychotic disorders. All patients were antipsychotic-naïve at the moment of the study. Exclusion criteria included: presence of any concomitant medical or neurological illness, current substance abuse or history of substance dependence (excluding nicotine), comorbidity of any other psychiatric disorder, and psychomotor agitation. The Positive and Negative Syndrome Scale (PANSS)^47^ was used to assess symptomatology severity in the FEP group. Also, neurocognition was explored using the MATRICS Cognitive Consensus Battery (MCCB)^48^.

A healthy control group (HC, *n* = 13) included participants similar in age and gender to the FEP group. They were recruited from schools and social networks advertisements. Additional exclusion criteria for HC participants were having previous or present psychiatric illness, as revealed by the Symptom Check List 90 (SCL 90)^49^ and/or positive familiar history for psychosis. All participants were screened for drug abuse (e.g., cannabis, cocaine, heroin, opioids, and benzodiazepines) at the time of study inclusion.

The study was approved by the Ethics and Scientific Committees of the INNN and subjects signed an informed consent if they agreed to participate in the study. Written consent from both parents and assent for subjects younger than 18 years of age (the age of consent in Mexico) was obtained from all subjects prior to participation.

### Experimental paradigm

The experimental task was originally proposed by Schröger and Wolff^50–52^ and has been used by our group^53–55^ to evoke the so-called Distraction potential, which includes the Mismatch Negativity (MMN), the P3a, and the Reorientation Negativity (RON)^5^. Given our interest in P3a, we focused on this ERP and its underlying oscillatory activity. The task consisted of an auditory involuntary attention task, with the administration of pure frequent tones (90%, 1000 Hz) and pure deviant tones (10%, 900, and 1100 Hz) delivered binaurally through earphones, with an intensity of 80 dB. The tones were presented in a pseudo-random order, in such a way that two deviant tones were never presented subsequently. All tones had two durations with the same probability of presentation: 200 and 400 ms. The inter-stimulus interval was 1500 ms.

Participants remained in a chair in an acoustically attenuated room and were asked to distinguish short (200 ms) from long (400 ms) tones and to respond as quickly as they could regardless of the tone pitch, by pressing one of two buttons of a response panel. A total of 798 tones were delivered (717 frequent, 40 low deviants (900 Hz), and 41 high deviants (1100 Hz)). Before performing the task, all the participants were trained with one block of 30 frequent tones. In order to be included in the study, they had to respond correctly on 60% of the training block. All participants responded with the right hand and with closed eyes. Reaction times (RT) and hit rates (HR) were estimated along the task.

### EEG recording and analysis

A digital monopolar EEG with an online bandwidth of 0.5–40 Hz and a sampling rate of 1000 Hz was continuously recorded with SCAN 4.3.1 software (Neuroscan Inc., Charlotte, North Carolina), using a NuAmps digital amplifier (Neuroscan Inc., Charlotte, North Carolina). Linked ear lobes were used as the reference. We used 19 tin electrodes (10-20 International System^56^) attached to an elastic cap (ElectroCap Inc., Eaton, Ohio). The electrode impedance was kept below 5 KΩ. All EEG were recorded between 9 am and 1 pm. This schedule coincided with usual patients’ appointments at the INNN and allowed us to reduce excessive fatigue in our participants.

All data analyses were done using Matlab custom scripts and the Fieldtrip toolbox^57^. After preprocessing EEG segments (-1 to 1 s respecting stimuli onset) and resampling to 256 Hz, those showing ±50 µV artifacts in any electrode were excluded from the analyses. Also, epochs with incorrect responses or responses given 200 ms before or 1100 ms after tone onset were eliminated from further analysis, as well as those corresponding to frequent tones present after a deviant tone. In addition, epochs containing eye blinks were excluded. The remaining epochs underwent visual inspection to exclude those with muscle artifacts. Finally, demean, baseline correction (-200 to 0 ms before stimuli onset), and linear detrend were applied to all epochs.

### EEG oscillations

We explored EEG oscillations in terms of power, phase-locking to the tones, and power-based connectivity. Time-frequency representations (TFR) of power in all channels were performed. We used a fast Fourier transformation (FFT) with an adaptive sliding time window of two cycles long (ΔT = 2/f; e.g. ΔT = 200 ms for 10 Hz) in steps of 10 ms from 1 to 30 Hz. As the frequency sample we used was 256 Hz (maximum detectable frequency=128 Hz), the number of points for each frequency was 2*256/f (e.g. 51 pts. for 10 Hz). A Hanning taper (ΔT long) was multiplied by the data prior to the FFT. The power of the individual trials was averaged over stimuli type (frequent and deviants) and log-transformed.

In order to explore the phase-locking after stimuli onset across trials, phase data were used to estimate the phase-locking factor (PLF) or inter-trial phase clustering^58^. The PLF over N trials is defined in Eq. (1):

1 $$PLF\left( {f_o,t} \right) = \;\frac{1}{N}\left| {\mathop {\sum }\limits_{k = 1}^N e^{i\varphi ^k(f_0,t)}} \right|$$

where *φ*^*k*^*(f*_*0*_*, t)* corresponds to the estimated phase at frequency *f*_*0*_ and time t resulting from the time-frequency analysis. The PLF represents the extent to which the distribution of phase angles at each time-frequency-sensor point across trials is non-uniformly distributed. A PLF close to 0 reflects a strong phase variability, whereas a PLF=1 indicates that all trials exhibit the same phase at a given frequency and time point. As for the TFR analysis of power, the PLF was calculated with respect to a sliding time window 2 cycles long to which we applied a Hanning taper. Obtained PLF values were normalized by transforming them to Rayleigh Z values (Z=n·PLF^2^, where n is the number of trials^59^).

Detection of auditory infrequent or novel stimuli has been reported to increase the activity of fronto-parietal regions associated with the ventral attention network (VAN), as revealed by the modulation of theta activity in healthy participants^33^. Therefore, we explored the TFR of connectivity between ipsilateral frontal and parietal channels close to inferior frontal regions and the temporo-parietal junction, which are part of the VAN (i.e. F7-P7 and F8-P8), by calculating the correlation of power evoked by the tones. Power-based connectivity is a recommended method for exploratory analysis due to its flexibility, and is the most similar measure to neural communication as expressed by functional magnetic resonance (fMRI) methods^59^. Power-based connectivity values were based on Pearson coefficients and were transformed to Fisher-Z values before entering statistical analyses.

### P3a

**Fig. 3:**
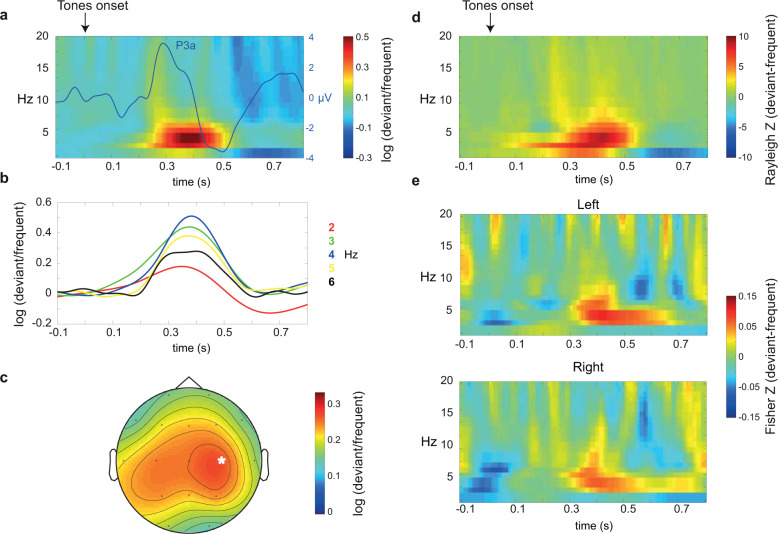
Time-frequency windows of interest across all participants. **a** TFR of power difference between deviant and frequent tones, showing modulation of theta oscillations. The P3a ERP (deviant-frequent) is shown superimposed, with voltage scale at the right *y* axis. **b** Maximal differences of power were found at 4 Hz. **c** Maximal theta (4 Hz) difference in power was found at right central regions (C4, marked with an asterisk). **d** TFR of phase alignment (PLF) difference between tones, with similar results to TFR of power. **e** TFR of fronto-parietal connectivity difference between deviant and frequent tones. The upper panel shows the left pair (F7-P7) and the lower panel shows the right pair (F8-P8).

Finally, the P3a was estimated as a time-domain correlate of evoked theta activity. Averages in the time domain were obtained separately for frequent and deviant tones. The P3a was considered as the maximum voltage peak between 250 and 500 ms in the infrequent tones’ waveform (Fig. 3a). Mean voltage values with respect to the peak from the deviant and frequent tones’ waveform were extracted for comparisons between tones and groups. Across all participants, the P3a showed maximal amplitude difference at C4 channel (similar to theta oscillations, see Time-frequency windows of interest section), so mean amplitudes at this region were used for comparison between groups.

In order to guarantee similar signal to noise ratios between tone types, the same number of trials for each one was used within participants for all the analyses (TFR, PLF, connectivity, and ERP). In each participant, we matched the number of trials by selecting randomly the same number of frequent trials as the number of accepted deviant trials. We chose to do this because the ERP and PLF values are sensitive to the number of observations^59^. The mean number of included trials for each type of tone across participants was 64 ± 15, with significant differences between groups (t_(26)_=2.24, *p*=0.03), given the smaller number of correct responses in the FEP group (see Results). Therefore, we included this variable as covariate in all statistical comparisons between groups. We chose to follow this method rather than reducing the number of trials in the HC group, given that this approach reduces statistical power and increases the Type II error rate (likelihood of accepting the null hypothesis when it is false). Moreover, unbalanced number of trials does not increase the Type I error rate (likelihood of rejecting the null hypothesis when it is true), especially when using mean values (across time and/or frequency), which was our case^4^.

### Time-frequency windows of interest

In order to identify time-frequency windows of interest, we followed a data-driven approach including two main steps: (1) As temporal dynamic of novelty detection was the main objective of this study, we averaged the TFR of total power across all participants (regardless of group) and computed the difference between tones (log(deviant/frequent)). This contrast revealed increased theta (4–7 Hz) power at central regions for deviant compared to frequent tones and on the time range usually described for the P3a^7,54,55^, i.e. from 300 to 500 ms after tone onset (Fig. 3a). By exploring the theta increase in each frequency bin between 2 and 6 Hz, the maximal amplitude was found in 4 Hz (Fig. 3b) and in the C4 channel (Fig. 3c). Therefore, mean values from C4 in this time-frequency window (300–500 ms, 4 Hz) were used for subsequent comparisons between groups. PLF showed a similar time-frequency distribution (Fig. 3d), so mean values were extracted with the same time-frequency-channel window criteria as for power. 2) Power-based connectivity contrasts between deviant and frequent tones revealed increased values in the theta range as well for deviant vs. frequent tones between bilateral fronto-parietal regions (Fig. 3e). Mean connectivity values from each hemisphere in the same time-frequency windows as for power and PLF were used for subsequent comparisons between groups. By identifying channel and time-frequency windows of interest not based on apparent differences between groups, but between tone types (deviant vs. frequent), we avoided committing a circular inference error (i.e. running statistical comparisons based on a priori observed differences between groups^59^).

### Statistics

Descriptive analysis was performed in terms of mean, standard deviation, and percentages. Demographic, clinical, and neuropsychological variables were compared by t-tests or Chi-square tests as required.

The behavioral variables (RT and HR) were analyzed with repeated measures ANOVA (RM-ANOVA), with type of tone (frequent/deviant) as within-subject factor, and group (FEP/HC) as between-subject factor.

For power and PLF, theta values from each tone type were analyzed with a RM-ANOVA, with tone type as within-subject factor and group as between-subject factor. A similar RM-ANOVA was used to compare the P3a between tones and groups. In the case of power-based connectivity, we included the side (left/right) and tone type as within-subject factors, and group as between-subject factor. Statistical significance was set at *p* ≤ 0.05 for all RM-ANOVA. *Post hoc* comparisons were made using the Bonferroni test. Since time-frequency windows were selected based on differences between tone type, we focused on group effects or on tone by group (and tone by side in the case of connectivity) interactions.

Finally, Pearson correlation analyses between EEG measures (i.e. power, PLF, connectivity, or P3a) and clinical variables (i.e. PANSS subscores and general score, and MCCB total score) were performed exclusively using the electrophysiological variables that proved to be different in the FEP group. For the analyses including the PANSS, significance was established under a Bonferroni correction for multiple correlations (alpha = 0.05/PANSS scores (4) = 0.0125). IBM SPSS 20 software (IBM Corp.) was used for all statistical analyses.

### Reporting summary

Further information on research design is available in the Nature Research Reporting Summary linked to this article.

## Supplementary information

Reporting summary

## Data Availability

The data that support the findings of this study are available from the corresponding author upon reasonable request.

## Code Availability

The MATLAB code that supports the findings of this study are available from the corresponding author upon reasonable request.
